# Supplementary material for: A kinematic synergy for terrestrial locomotion shared by mammals and birds
Source: eLife. 2018 Oct 30;7:e38190. doi: 10.7554/eLife.38190 (PMC6257815; doi:10.7554/eLife.38190)
Supplement: Figure 1—source data 1. [file elife-38190-fig1-data1.zip › SourceData1-Figure1/readme_datafile.pdf]

Once opened the **data.mat** file in a Matlab workspace, the file is a struct, named 'ELIFEdati' with the fields:

- sbj - it contains the subject's data substructures. Its fields are: forelimb, hindlimb, and timing, that are Nt x 1 structs, with Nt equals to the number of trials (i.e. video recorded). The forelimb and hindlimb substructures are organized with the following fields:
    - fname
    - idanimale: identification number for each animal and trial (i.e. video recorded)

  The timing substructure is organized with the following fields:
    - name: file name
    - PL: phase lag matrix
    - idanimale: identification number for each animal and trial (i.e. video recorded)

- sbjlabel: labels for each animal, sorted as they are presented

- motionlabel: label of the motion. In this case, walking.

- ROM: this structure contains the following field
    - gambe: limb label as they are sorted in the structure
    - angolilabel: labels for each angle as they are sorted in the structure
    - angoli: 1x2 cell, one for hindlimb and one for forelimb, as labeled in ROM.gambe. Each cell contains a AxJ cell, where A corresponds to the number of angles labelled in ROM.angolilabel, and J = total number of animals. In each cell, the averaged elevation angles that were used for the analyses, see Material and Methods section.
    - PCAordinata: another nested substructure that contains the variables output of the Principal Component Analyses. It comprises two subfield (hindlimb and forelimb), that are substructures with the field:
        - labelangoli (angles label for which the PCA analyses is done);
        - angoli (averaged angles over all strides for each species);
        - PV (eigenvector percent of variance for each species);
        - Unew (eigenmatrix for each species with eigenvectors in columns);
        - eigenvectorstat (eigenvectors values – see *);
    - Ns: Stride Numbers Ns{1,gg}{jj,1} = number of strides for the jj^{th} animal with jj = 1, …, J and gg=1 for hindlimb, and gg=2 for forelimb.
    - Colore: struct with subfield – 1) 'color2', the color used in all the figures; 2)'markertype', the marker for every figure; 3)'markerid', identifier for each animal; 4)OrderLabel is a 54x6 cell containing the sbjlabel as they are listed in this structure, the class, the markertype used in each figure, the Order and the corresponding scientific name for each animal

* eigenvectorstat: substructure containing the mean vector (VM) of the eigenvector listed in AA, which is J x 3 matrix, J = total number of animals, 3 = dimension of the space. H is the matrix used for the rotation of the eigenvectors as explained below.

Let $(aa_1, aa_1, aa_1), \ldots, (aa_J, aa_J, aa_J)$ be the direction cosines of the sth eigenvectors listed in eigenvectorstat(1,s).AA, where J is the total number of species. These points lay on the unit sphere. The location of these points can be summarized by their sample mean vector ($\acute{x}$,$\acute{y}$,$\acute{z}$)=eigenvectorstat(1,s).VM, which is defined as:$(\acute{x}, \acute{y}, \acute{z}) = (\sum x_i, \sum y_i, \sum z_i)$, where the sum is over the number of points J. In Fig 4 (top panels) each vector u_3 = eigenvectorstat(1,3).AA(j,:), j=1,…,J yields 1 point corresponding to the projection of the normal to the plane onto the unit sphere, the axes of which are the direction cosines with the axes of segment elevation angles (u_{3t}, u_{3s}, u_{3f}). The u_3 vectors for different animals lay on the plane perpendicular to the averaged u_1 vector (eigenvectorstat(1,1).VM) across animals. We computed the rotation matrix H using the azimuth and elevation angles of the averaged u_1 vector across animals. Then AAruot, VMruot are the value of the rotated matrix AA and the rotated vector VM, and azimuthruot and elevationruot are respectively the azimuth and elevation angles of the vectors in AAruot. The α angles of rotation of the u_3 vectors on this plane that were computed in our analyses and displayed in Figures are in eigenvectorstat(1,3).azimuthruot
