# Supplementary material for: A kinematic synergy for terrestrial locomotion shared by mammals and birds
Source: eLife. 2018 Oct 30;7:e38190. doi: 10.7554/eLife.38190 (PMC6257815; doi:10.7554/eLife.38190)
Supplement: Figure 1—source data 1. [file elife-38190-fig1-data1.zip › SourceData1-Figure1/readme.pdf]

The Source Data 1-Figure 1 folder contains the following files:

mfiles
    LocomotionCloud.m
        cerchio.m
        intersezionerettadisco.m
        traiettoriaEP.m
        trovaConfiguration.m
        calcola colori.m
        trovaTheta1eTheta2.m
    hindlimbVSforelimbFORfigure1B.m
    hindlimbCamelVShindlimbFlamingoFORfigure1Bright.m
mat data
    data.mat
    Figure1Aforelimb.mat
    Figure1Ahindlimb.mat
    Figure1BlefthindlimbVSforelimbCheetah.mat
    Figure1BrighthindlimbCamelVShindlimbFlamingo.mat

To reproduce the Figure1 panel A:
1. in Matlab set as working directory the folder Source Data 1-Figure 1
2. Run LocomotionCloud.m
3. Select the limb and press the OK button

To reproduce the Figure1 panel B left
1. Run hindlimbVSforelimbFORfigure1B.m

To reproduce the Figure1 panel B right
1. Run hindlimbCamelVShindlimbFlamingoFORfigure1Bright.m

Giovanna Catavitello - 18/10/2018
