# Supplementary material for: A kinematic synergy for terrestrial locomotion shared by mammals and birds
Source: eLife. 2018 Oct 30;7:e38190. doi: 10.7554/eLife.38190 (PMC6257815; doi:10.7554/eLife.38190)
Supplement: Figure 2—source data 1. [file elife-38190-fig2-data1.zip › SourceData2-Figure2/readme.pdf]

The Source Data 2-Figure 2 folder contains the following files:

mat data

Figure2ROM.mat

1. load('Figure2ROM.mat') in the Command Window
In the workspace you will find a table named ROM, with variable names: thigh, shank, foot, scapula, upper arm, lower arm, hand.

Giovanna Catavitello - 18/10/2018
