# Supplementary material for: A kinematic synergy for terrestrial locomotion shared by mammals and birds
Source: eLife. 2018 Oct 30;7:e38190. doi: 10.7554/eLife.38190 (PMC6257815; doi:10.7554/eLife.38190)
Supplement: Figure 2—figure supplement 2—source data 1. [file elife-38190-fig2-figsupp2-data1.zip › SourceData2-Figure2supplement2/readme.pdf]

The Source Data 2-Figure 2 supplement 2 folder contains the following files:

mat data
- Figure2supp2B.mat
- Figure2supp2C.mat
- Figure2supp2D.mat
- Figure2supp2E.mat
- Figure2supp2Fforelimb.mat
- Figure2supp2Fhindlimb.mat

load(‘Figure2supp2B.mat’) load Figure2supp2B table with variables: tFL and tFLcontr. See Figure 2-figure supplement 2B

load('Figure2supp2C.mat') load Figure2supp2C table with variables: HLstanceduration and FLstanceduration. See Figure 2-figure supplement 2C

load('Figure2supp2D.mat') load Figure2supp2D table with variable: strideduration. See Figure 2- figure supplement 2D

load('Figure2supp2E.mat') load Figure2supp2E table with variable: trunkinclination. See Figure 2- figure supplement 2E

load('Figure2supp2Fhindlimb.mat') load Figure2supp2Fhindlimb table with variables: horizexcurs and vertexcurs. See Figure 2- figure supplement 2F hindlimb panel

load('Figure2supp2Fforelimb.mat') load Figure2supp2Fforelimb table with variables: horizexcurs and vertexcurs. See Figure 2- figure supplement 2F forelimb panel

Giovanna Catavitello – 18/10/2018
