# Supplementary material for: A kinematic synergy for terrestrial locomotion shared by mammals and birds
Source: eLife. 2018 Oct 30;7:e38190. doi: 10.7554/eLife.38190 (PMC6257815; doi:10.7554/eLife.38190)
Supplement: Figure 3—source data 1. [file elife-38190-fig3-data1.zip › SourceData3-Figure3/readme.pdf]

The Source Data 3-Figure 3 contains the following files:

mat data
    Figure3.mat

load(‘Figure3.mat’) load PV3 table with variables: PV3HL, PV3FLupp, PV3FLlow. See Figure 3

Giovanna Catavitello – 18/10/2018
