# Supplementary material for: A kinematic synergy for terrestrial locomotion shared by mammals and birds
Source: eLife. 2018 Oct 30;7:e38190. doi: 10.7554/eLife.38190 (PMC6257815; doi:10.7554/eLife.38190)
Supplement: Figure 3—figure supplement 1—source data 1. [file elife-38190-fig3-figsupp1-data1.zip › SourceData3-Figure3supplement1/readme.pdf]

The Source Data 3-Figure 3 supplement 1 contains the following files:

mat data
- U3FLlow.mat
- U3FLupp.mat
- U3HL.mat

load('U3FLlow.mat') load UFL2 table with variables: U1, U2, U3, that corresponds to the components of the u_{3u}, u_{3l}, and u_{3h} vectors . See Figure 3-figure supplement 1

load('U3FLupp.mat') load UFL1 table with variables: U1, U2, U3, that corresponds to the components of the u_{3sc}, u_{3u}, and u_{3l} vectors . See Figure 3-figure supplement 1

load('U3HL.mat') load UHL table with variables: U1, U2, U3, that corresponds to the components of the u_{3t}, u_{3s}, and u_{3f} vectors . See Figure 3-figure supplement 1

Giovanna Catavitello - 18/10/2018
