# Supplementary material for: A kinematic synergy for terrestrial locomotion shared by mammals and birds
Source: eLife. 2018 Oct 30;7:e38190. doi: 10.7554/eLife.38190 (PMC6257815; doi:10.7554/eLife.38190)
Supplement: Figure 3—figure supplement 2—source data 1. [file elife-38190-fig3-figsupp2-data1.zip › SourceData3-Figure3supplement2/readme.pdf]

The Source Data 3-Figure 3 supplement 2 folder contains the following files:

mat data
- Fig3suppl2forelimblow.mat
- Fig3suppl2forelimbupp.mat
- Fig3suppl2hindlimb.mat

In each mat file the first column contains the the label for each subject as they are labelled in Figure 3 -figure supplement 2A. The second column contains the PC_1 and PC_2 variables. The third column contains the push off value (corresponding to the black line for each gait loop). The last column has the value for the panelB

load('Fig3suppl2forelimblow.mat') load Fig3suppl2forelimblow Matlab cell.

load('Fig3suppl2forelimbupp.mat') load Fig3suppl2forelimbupp Matlab cell.

load('Fig3suppl2hindlimb.mat') load Fig3suppl2hindlimb Matlab cell.

Giovanna Catavitello – 18/10/2018
