# Supplementary material for: A kinematic synergy for terrestrial locomotion shared by mammals and birds
Source: eLife. 2018 Oct 30;7:e38190. doi: 10.7554/eLife.38190 (PMC6257815; doi:10.7554/eLife.38190)
Supplement: Figure 4—figure supplement 1—source data 1. [file elife-38190-fig4-figsupp1-data1.zip › SourceData4-Figure4supplement1/readme.pdf]

The Source Data 4-Figure 4 supplement 1 contains the following files:

mat data
    Figure4supp1.mat
R file
    Figure4suppl.R
txt format file
    PhylogeneticTree_completo

load('Figure4supp1.mat') load PhyloazimuthHL table with variable Phyloazimuth, that is the azimuth computed after phylogenetic correction.
The R file contains the algorithm to reproduce the Figure 4- figure supplement 1. To be run in the R environment.
PhylogeneticTree_completo.txt contains the Taxonomic Tree to obtain Figure 4-figure supplement 1 in Newick format.

Giovanna Catavitello - 18/10/2018
