# Supplementary material for: A kinematic synergy for terrestrial locomotion shared by mammals and birds
Source: eLife. 2018 Oct 30;7:e38190. doi: 10.7554/eLife.38190 (PMC6257815; doi:10.7554/eLife.38190)
Supplement: Figure 5—source data 1. [file elife-38190-fig5-data1.zip › SourceData5-Figure5/readme.pdf]

The Source Data 5-Figure 5 contains the following files:

mat data

- Figure5.mat
- Figure5A.mat
- Figure5B.mat

load('Figure5A.mat') load Fig5A table with variables: FOOminusSHA and UPPminusSCA.
load('Figure5.mat') load PlaneOrientation table with variables: alphaHL and alphaFLupp

To obtain Figure 5A hindlimb panel: x=PlaneOrientation.alphaHL, y=Fig5A.FOOminusSHA
To obtain Figure 5A forelimb panel: x=PlaneOrientation.alphaFLupp,
y=Fig5A.UPPminusSCA

load('Figure5B.mat') load Tminima table containing minimum values of the following elevation angles: thigh, shank, foot, scapula, upperarm, lowerarm, and hand. See Figure 5B

Giovanna Catavitello - 18/10/2018
