# Supplementary material for: A kinematic synergy for terrestrial locomotion shared by mammals and birds
Source: eLife. 2018 Oct 30;7:e38190. doi: 10.7554/eLife.38190 (PMC6257815; doi:10.7554/eLife.38190)
Supplement: Figure 6—source data 1. [file elife-38190-fig6-data1.zip › SourceData6-Figure6/readme.pdf]

The Source Data 6-Figure 6 folder contains the following files:

mfiles
- Figure6A.m
    - faigriglia.m
    - PCA_sin_model_figure6A.m
- Figure6C.m

mat data
- data.mat
- Figure6A.mat
- Figure6C.mat

To reproduce the Figure6 panel A:
1. in Matlab set as working directory the folder Source Data 6-Figure 6
2. Run Figure6A.m

To reproduce the Figure6 panel C:
1. in Matlab set as working directory the folder Source Data 6-Figure 6
2. Run Figure6C.m

Giovanna Catavitello - 18/10/2018
